# The hidden burden: prevalence and risk factors of long COVID among university students in Chiang Mai, Thailand

**DOI:** 10.1186/s12889-025-25457-3

**Published:** 2025-11-24

**Authors:** Pheerasak Assavanopakun, Suphapich Wangkawong, Wuttipat Kiratipaisarl, Wachiranun Sirikul, Tharntip Promkutkao, Suchat Promkutkeo, Amornphat Kitro

**Affiliations:** 1 Department of Community Medicine, Faculty of Medicine, Chiang Mai University, Chiang Mai, 50200 Thailand; 2 Environmental and Occupational Medicine Excellence Center (EnOMEC), Faculty of Medicine, Chiang Mai University, Chiang Mai, 50200 Thailand; 3 Faculty of Medicine, Chiang Mai University, Chiang Mai, 50200 Thailand; 4 Department of Biomedical Informatics and Clinical Epidemiology, Faculty of Medicine, Chiang Mai University, Chiang Mai, 50200 Thailand; 5 School of Economics, Faculty of Economics, Chiang Mai University, Chiang Mai, 50200 Thailand

**Keywords:** COVID-19, Long COVID, Risk factor, University students, Thailand

## Abstract

**Background:**

As the COVID-19 pandemic transitions to an endemic phase, long COVID symptoms following SARS-CoV-2 infection have emerged as a new global health challenge. However, its impact on university students remains underexplored. This study aimed to assess the prevalence of long COVID symptoms and identify its predictive factors.

**Methods:**

A cross-sectional study was conducted from February to August 2023 among Thai university students in Chiang Mai. An online questionnaire collected data on demographics, COVID-19 history, vaccination, and health status. Multivariable binary logistic regression was used to identify factors associated with long COVID.

**Results:**

A total of 997 students participated (60.5% female, mean age 20.6 years). Of these, 60.9% had received at least three COVID-19 vaccine doses, and 21.4% had received more than three mRNA vaccine doses. The prevalence of long COVID symptoms was 21.9% (*n* = 218). Common symptoms included respiratory issues (54.6%), neurological complaints (50.4%), psychological symptoms (42.7%), and poor sleep quality (34.9%). Significant predictors of long COVID included severe initial infection (aOR = 15.3; 95% CI: 5.3–44.3; *p* < 0.001), longer illness duration (aOR = 1.05; 95% CI: 1.0-1.1; *p* = 0.031), and poor sleep quality (aOR = 2.1; 95% CI: 1.4–3.1). Each additional dose of mRNA vaccine reduced the likelihood of severe outcomes by 14% (aOR = 0.86; 95% CI: 0.8-1.0; *p* = 0.044).

**Conclusion:**

A minority of Thai university students reported long COVID symptoms. Vaccination, especially with multiple mRNA doses, was linked to reduced risk. Early detection and targeted support during recovery may help mitigate long-term health consequences in this group.

**Supplementary Information:**

The online version contains supplementary material available at 10.1186/s12889-025-25457-3.

## Introduction

Since the World Health Organization (WHO) declared COVID-19 a global pandemic on March 11, 2020 [1] The world has faced profound and lasting health challenges. Although many countries have transitioned from a pandemic to an endemic phase, a significant concern remains: the persistence of symptoms long after the acute infection has resolved [2]. This condition, commonly referred to as “long COVID,” “long-term COVID,” or “post-acute sequelae of SARS-CoV-2 infection (PASC),” has emerged as a growing public health issue [3, 4]. Typically, the viral load becomes undetectable within a few weeks after diagnosis, marking the end of the acute phase and the beginning of the post-acute period [2]. However, many individuals continue to experience a range of physical, neurological, and psychological symptoms beyond this point, highlighting the long-term impact of SARS-CoV-2 infection on multiple organ systems [5]. Understanding long COVID is essential for effective prevention, management, and support strategies in the ongoing response to the pandemic.

Long COVID can manifest in a variety of ways, including neuropsychiatric, cardiorespiratory, gastrointestinal, dermatologic, and other symptoms [6]. The incidence of symptoms such as fatigue and headache, as well as other factors associated with long COVID among children and adolescents, was reported worldwide [7], even though there were a few reports in Thailand. Several studies have found that being female, having more than five early symptoms, having severe acute COVID-19, and having few previous COVID-19 vaccines or boosters are some of the most important higher-risk factors for long COVID [8, 9]. One previous study found that age, underlying allergy diseases, and residing in polluted locations may be related to long COVID, although it was conducted in young children [10]. Some children and adolescents with COVID-19 had long-term infections and disabilities, while many experienced symptoms despite testing negative for the virus [11]. Immunization is another key to large-scale disease prevention. Immunizing a large section of the population can boost community immunity through herd immunity. If vaccine coverage is high enough, it can prevent the disease from spreading [12, 13]. There might be an alteration in strong health measurements since COVID-19 has been downgraded from a communicable disease to a communicable disease under surveillance [14].

Evidence on the prevalence of long COVID-19 symptoms among adolescents in Thailand, particularly university students, remains limited. This study aims to assess the prevalence of long COVID among university students in Chiang Mai, Thailand, and to identify factors associated with the development of long COVID symptoms in this population. The study offers a novel contribution by focusing on university students. The findings may inform the development of targeted interventions for early symptom detection and timely management by the public health sector and relevant stakeholders, emphasizing prevention and appropriate care for individuals with prior COVID-19 infection or those at risk of developing Long COVID. Moreover, the results can guide school- and university-based strategies to design health support systems and learning platforms that are responsive to students’ educational needs and well-being in the post-COVID era.

## Methods

### Study design, study population, and sample size estimation

**Fig. 1:**
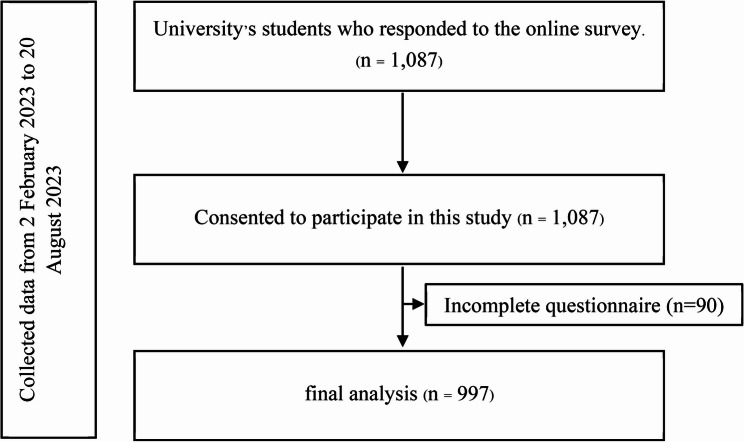
Study Flow Diagram

This cross-sectional study was conducted between February and August 2023 using an online survey targeting undergraduate students from seven universities in Chiang Mai, Thailand—the country’s second-largest province, with a total university student population of 83,877. Data were collected via REDCap (Research Electronic Data Capture), a secure, web-based platform designed for managing research data [15, 16]. Before data collection, the research team contacted university administrators and obtained approval letters from the deans of each institution. During the data collection phase, we collaborated with university COVID-19 prevention teams to explain the study’s objectives, procedures, and data collection methods. Designated university coordinators facilitated the distribution of the online survey to students (Fig. 1). To ensure confidentiality, coordinators were informed that they would not have access to the survey responses or the identities of participating students. Participation was entirely voluntary and had no impact on students’ academic standing. The definition of Long COVID in this study was followed the UK National Institute for Health and Care Excellence (NICE), which defines “Long COVID” as the presence of persistent symptoms lasting 4 weeks or more after acute infection, in the absence of an alternative etiological diagnosis [17].

The required sample size was estimated using the finite population prevalence estimation method, assuming a 9.1% prevalence of post-infection symptoms at three months, with a 2% absolute error margin (d) and a 0.05 alpha level. This yielded a minimum required sample size of 787 participants [18, 19].

### Questionnaire design

The questionnaire for this study was developed and disseminated by the Ministry of Public Health of Thailand for direct use by medical personnel [20, 21]. The questionnaire was divided into four main sections:

1. Project Details and Consent: Information about the study and a consent form for participants.
2. Personal and Sociodemographic Information: This includes details such as age, gender, year of study, university affiliation, and type of discipline.
3. COVID-19 History and Health Data: This covers self-reported COVID-19 infections, vaccination history, treatments received, and post-infection symptoms. The post-infection symptoms were classified according to the organ system. The symptom categories included ten organ systems: respiratory, psychiatric, neurological, musculoskeletal, cardiovascular, dermatological, otolaryngological, immunological, gastrointestinal, and reproductive. Furthermore, nonspecific symptoms and sleep quality were distinct categories incorporated in this section.
4. Activities and Preventive Behaviors: Assesses participants’ behaviors after the “new normal” period, including mask usage when outdoors, use of alcohol-based hand sanitizers, and social distancing practices. Responses were categorized as “never,” “sometimes (occasionally practiced), or “always (practice that behavior regularly every time there is a risk of virus exposure).

An assessment of the qualitative questionnaire’s content validity was performed following the adaptation of the Ministry of Public Health’s questionnaire, created in conjunction with the Health Promotion Foundation. It underwent a content validity review by three experts, including a specialist in COVID-19 control and prevention from the Chiang Mai Provincial Public Health Office, a public health expert from Chiang Mai University, and a research instrument specialist from Chiang Mai University. The Cronbach’s alpha coefficient values for all sections of the questionnaire were above 0.7, ranging from 0.713 for the respiratory system to 0.942 for behavioral practices. Details for each subdomain are provided in the Supplementary Material (Table S1).

### Statistical analysis

All statistical analyses were performed using the statistical software STATA (Stata Corp. 2019, Stata Statistical Software: Release 16, Stata Corp LLC, College Station, TX, USA). For the categorical data, the personal information, details of outside activities, preventive or risky behaviors, willingness for vaccination, and COVID-19 information resources were described by a frequency and a percentage (n (%)). The continuous variables were described using a mean with a standard deviation (SD) for parametric data or a median with an interquartile range (IQR) for non-parametric data. A comprehensive exploratory analysis using a multivariable binary logistic regression was conducted to identify factors associated with long COVID-19 at one month; robust variance correction was used to address potential outliers and influential observations. Covariates for inclusion in the regression models were selected based on a well-established theoretical framework aligned with the research objectives and hypotheses. Variance inflation factor (VIF) was explored to examine the collinearity of determinants included in the statistical model. The threshold VIF of more than 10 indicated the risk of collinearity. The study findings were reported by the STROBE (Strengthening the Reporting of Observational Studies in Epidemiology) reporting guideline [22]. All statistical analyses were performed on a two-tailed basis, and a p-value of less than 0.05 was regarded as statistically significant.

### Ethical considerations

This study was approved by the Research Ethics Committee, Faculty of Medicine, Chiang Mai University, Thailand (Study Code: COM-2565–09280). Informed consent was obtained from all subjects involved in the study.

## Result

A total of 1,087 participants were recruited and consented to participate in the study, of whom 997 were included in the analysis. Ninety participants were excluded due to incomplete questionnaire. Among the 997 participants, the majority were female (60.5%, *n* = 603) with a mean age of 20.6 years (SD = 2.1). Most were affiliated with government universities (80.6%, *n* = 804), and nearly half (46.3%, *n* = 462) studied health and life sciences.

Regarding COVID-19 infection, 21.9% (*n* = 218) reported experiencing Long COVID symptoms, while 56.1% (*n* = 559) had symptoms lasting less than one month. The severity of infection varied: most participants reported minimal symptoms (34.9%, *n* = 348), followed by moderate symptoms (28.4%, *n* = 283), asymptomatic cases (12.3%, *n* = 123), and a small proportion reported severe symptoms (2.3%, *n* = 23). The median duration of treatment for previously infected participants was 8 days (IQR: 7–10), with those experiencing Long COVID having a longer median treatment duration of 10 days (IQR: 7–14).

COVID-19 vaccination uptake was high overall: 98.7% (*n* = 984) had received at least one dose, with 60.9% (*n* = 599) having received three or more doses. However, only 21.4% (*n* = 208) had received three or more doses of an mRNA vaccine. Among participants who had never been infected with COVID-19, vaccine coverage was slightly lower at 95.0% (*n* = 209), with 63.2% (*n* = 132) receiving at least three doses and 82.0% (*n* = 168) receiving fewer than three doses of an mRNA vaccine.

Preventive practices varied among participants. Overall, 56.3% (*n* = 561) reported always wearing a face mask outdoors, 51.6% (*n* = 514) sometimes used alcohol gel, and 53.8% (*n* = 536) sometimes practiced social distancing. Notably, participants who had never been infected with COVID-19 demonstrated higher adherence to preventive measures: 85.9% (*n* = 189) always wore a mask, 65.5% (*n* = 144) always used alcohol gel, and 55.0% (*n* = 121) always practiced social distancing. (Table 1)

**Table 1:** Sociodemographic characteristics of university students reporting long COVID Symptoms, Non-long COVID Symptoms, and those never infected with COVID-19

Parameters		Total	Long COVID	Non-Long COVID	*P*-value¤	Never got infected	*P*-value†
		*N* = 997	*N* = 218	*N* = 559		*N* = 220	
Sex	Male	394 (39.5%)	88 (40.4%)	233 (41.7%)	0.75	73 (33.2%)	0.14
	Female	603 (60.5%)	130 (59.6%)	326 (58.3%)		147 (66.8%)	
Mean age (years)		20.6 (2.1)	20.9 (2.9)	20.6 (1.6)	0.030*	20.3 (2.1)	0.016
Academic year	Year 1	181 (18.2%)	20 (9.2%)	103 (18.4%)	0.002*	58 (26.4%)	< 0.001**
	Year 2	304 (30.5%)	78 (35.8%)	159 (28.4%)		67 (30.5%)	
	Year 3	232 (23.3%)	65 (29.8%)	132 (23.6%)		35 (15.9%)	
	Year 4 and above	280 (28.1%)	55 (25.2%)	165 (29.5%)		60 (27.3%)	
Type of discipline	Science and technology	212 (21.3%)	39 (17.9%)	115 (20.6%)	0.49	58 (26.4%)	0.094
	Health and life sciences	462 (46.3%)	110 (50.5%)	256 (45.8%)		96 (43.6%)	
	Humanities	323 (32.4%)	69 (31.7%)	188 (33.6%)		66 (30.0%)	
Ever have a COVID-19 vaccination	Received COVID-19 vaccine	984 (98.7%)	216 (99.1%)	559 (100.0%)	0.078	209 (95.0%)	0.021*
	Not received COVID-19 vaccine	13 (1.3%)	2 (0.9%)	0 (0.0%)		11 (5.0%)	
The total number of vaccines received	< 3 doses	385 (39.1%)	76 (35.2%)	232 (41.5%)	0.12	77 (36.8%)	0.76
	≥ 3 doses	599 (60.9%)	140 (64.8%)	327 (58.5%)		132 (63.2%)	
The number of mRNA vaccines received	< 3 doses	763 (78.6%)	169 (80.5%)	426 (76.6%)	0.28	168 (82.0%)	0.71
	≥ 3 doses	208 (21.4%)	41 (19.5%)	130 (23.4%)		37 (18.0%)	
Infection severity	Asymptomatic	123 (12.3%)	14 (6.4%)	109 (19.5%)	< 0.001**	0 (0%)	N/A
	Minimal symptoms	348 (34.9%)	74 (33.9%)	274 (49.0%)		0 (0%)	
	Moderate symptoms	283 (28.4%)	115 (52.8%)	168 (30.1%)		0 (0%)	
	Severe symptoms	23 (2.3%)	15 (6.9%)	8 (1.4%)		0 (0%)	
Duration of Treatment in days, median (Q1, Q3)		8 (7, 10)	10 (7, 14)	7 (5, 10)	< 0.001**	N/A	N/A
Wearing mask when going outdoors	Always	561 (56.3%)	100 (45.9%)	272 (48.7%)	0.021*	189 (85.9%)	< 0.001**
	Sometimes	392 (39.3%)	113 (51.8%)	250 (44.7%)		29 (13.2%)	
	Not done	44 (4.4%)	5 (2.3%)	37 (6.6%)		2 (0.9%)	
Alcohol gel usage	Always	429 (43.0%)	85 (39.0%)	200 (35.8%)	0.005*	144 (65.5%)	< 0.001**
	Sometimes	514 (51.6%)	129 (59.2%)	317 (56.7%)		68 (30.9%)	
	Not done	54 (5.4%)	4 (1.8%)	42 (7.5%)		8 (3.6%)	
Social distancing	Always	336 (33.7%)	61 (28.0%)	154 (27.5%)	0.77	121 (55.0%)	< 0.001**
	Sometimes	536 (53.8%)	130 (59.6%)	324 (58.0%)		82 (37.3%)	
	Not done	125 (12.5%)	27 (12.4%)	81 (14.5%)		17 (7.7%)	

*p-value < 0.05, **p-value < 0.001

¤Comparing between long-COVID and non-long COVID group

†Comparing between long COVID and never get infected group

Among participants with long COVID symptoms, 19.7% (*n* = 43) experienced non-specific symptoms, while 54.6% (*n* = 119) reported respiratory symptoms such as shortness of breath and cough. Psychiatric symptoms were present in 42.7% (*n* = 93), and 50.4% (*n* = 108) had neurological symptoms. Additionally, 34.9% (*n* = 76) reported bad sleep quality and 23.9% (*n* = 52) experienced musculoskeletal symptoms, 21.6% (*n* = 47) had cardiovascular symptoms, and 20.2% (*n* = 44) reported dermatological symptoms. Among female participants, 42.3% (*n* = 58) experienced reproductive-related symptoms (Table 2).

**Table 2:** Prevalence of long COVID-19 symptoms among university students by affected organ systems (*n* = 218)

Long COVID symptoms are characterized by organ system	Symptom presented
Non-specific	43 (19.7%)
Respiratory	119 (54.6%)
Neurological	108 (50.4%)
Psychiatric	93 (42.7%)
Poor Sleep quality	76 (34.9%)
Musculoskeletal	52 (23.9%)
Cardiovascular	47 (21.6%)
Dermatological	44 (20.2%)
Otolaryngological	41 (18.8%)
Immunology	31 (14.2%)
Gastrointestinal	30 (13.8%)
Reproductive (*n* = 137, female)	58 (42.3%)

As shown in Table 3, three factors were significantly associated with a higher likelihood of long COVID-19 symptoms among university students, including infection severity, duration of treatment, and sleep quality. The severity of infection played a significant role, students with severe symptoms were 15.3 times more likely to experience long COVID (95% CI: 5.29–44.38, *p* < 0.001), those with moderate symptoms had a 6.2 times higher likelihood (95% CI: 3.12–12.38, *p* < 0.001), and those with minimal symptoms had a 2.2 times higher likelihood (95% CI: 1.13–4.33, *p* = 0.021) compared to asymptomatic individuals. Students who underwent a longer treatment duration had a higher likelihood of experiencing long COVID symptoms (aOR = 1.05, 95% CI: 1.00–1.09.00.09, *p* = 0.031). Poor sleep quality was also a significant factor, with those experiencing poor sleep having a 2.1 times higher likelihood of long COVID symptoms (95% CI: 1.41–3.09, *p* < 0.001) compared to those with normal sleep quality. Conversely, receiving an mRNA vaccine was associated with a lower risk of long COVID symptoms (aOR = 0.86, 95% CI: 0.75–1.00.75.00, *p* = 0.044). No collinearity was detected based on mean VIF of 1.11 with determinant-specific values ranging from 1.04 for age to 1.14 for days under treatment.

**Table 3:** Factors associated with long COVID-19 among university students

Parameter	Adjusted Odds Ratio	95% Confidence Interval		*P*-value	VIF***
		Lower bound	Upper bound		
Gender					
Male (ref: female)	1.23	0.86	1.76	0.251	1.14
Age (per 1 year increase)	1.06	0.96	1.18	0.255	1.04
Infection severity					
Asymptomatic	ref				1.07
Minimal symptoms	2.21	1.13	4.33	0.021*	
Moderate symptoms	6.22	3.12	12.38	< 0.001**	
Severe symptoms	15.32	5.29	44.38	< 0.001**	
Days under treatment (per 1-day increase)	1.05	1.00	1.09	0.031*	1.14
Sleep Quality					1.08
Poor (ref: no symptom)	2.09	1.41	3.09	< 0.001**	1.07
mRNA (per 1 dose increase)	0.86	0.75	1.00	0.044*	1.11

*p-value < 0.05, **p-value < 0.001, *** Mean VIF = 1.11

## Discussion

To the best of our knowledge, this study found that the prevalence of long COVID among university students was 21.9%. The most reported symptoms were respiratory (54.6%), followed by neurological (50.4%), psychological (42.7%), and poor sleep quality (34.9%). Factors associated with a higher risk of long COVID included a history of moderate to severe symptoms, longer treatment duration, and poor sleep quality. In contrast, receiving an mRNA COVID-19 vaccine was associated with a lower risk of long COVID symptoms.

Our study found the prevalence of long COVID at 21.9%, which was slightly lower than the pooled prevalence of 23.4% among children and adolescents under 18 years of age reported in a 2023 meta-analysis, in which approximately one-fourth of the included studies were conducted among Asian populations [7]. Our finding was also substantially lower than the global adult prevalence of 43% and the 51% among Asian adults [23]. Additionally, it was lower than a study conducted in a U.S. university setting, which reported a prevalence of 36% [8] Prior research in Thailand during 2021–2022 reported long COVID prevalence rates of 32.9%−60% among adolescents and adults in Bangkok [24, 25]. Several factors may explain why our study reported a lower prevalence of long covid compared with previous studies. Younger and healthier individuals may have a lower risk, consistent with reports showing a lower incidence among children (4%) than adults (10.2–25.9%) [8, 26]. In addition, differences in the circulating SARS-CoV-2 variants during the post-pandemic period may also have contributed to the reduced risk [27]. Although the risk of long COVID was lower, it was not negligible. Therefore, university students should be advised to monitor their health for at least one month following COVID-19 diagnosis, especially those with a history of moderate to severe symptoms at diagnosis could increase the risk by 15 times, as well as a longer duration of treatment, and seek appropriate medical care if any symptoms develop.

The top three organ systems most affected in our study were the respiratory system (54.6%), the neurological system (50.4%), and the psychiatric system (42.7%). Our findings are consistent with those from a study conducted in a U.S. university setting, which reported respiratory symptoms such as cough and shortness of breath in 43.2–64.5% of participants, and a slightly higher rate of neurological symptoms, including headache and fatigue, ranged from 48.9 to 52.3% [8]. Moreover, a meta-analysis among adolescents reported a lower prevalence of long COVID symptoms, with respiratory symptoms at 14.8%, neurological symptoms at 13.5%, and psychiatric symptoms at 12.3%, respectively [7]. A 2022 review similarly found a lower rate of neurological post-COVID symptoms, including fatigue (37%), brain fog (32%), and headache (10%), with some symptoms persisting for over 24 weeks [28, 29]. The mechanisms by which symptoms develop throughout multiple systems of the body tend to involve tissue damage attributable to immune-mediated responses and inflammation. Furthermore, neurological and psychological manifestations may result from impaired neurological signaling [30]. Although post COVID manifestations vary widely, respiratory and neuropsychiatric symptoms should receive particular attention due to their potential to cause long-term sequelae. Raising awareness among university students about the possible burden and impacts on daily life is essential for early recognition and appropriate management. Moreover, healthcare providers should schedule follow-up evaluations for high-risk individuals to facilitate early detection.

Our study found that 34.9% of participants reported poor sleep quality. This prevalence is comparable to that reported in a meta-analysis, which found sleep disturbance in 30.7% (95% CI 19.3–42.1%), with symptoms persisting for more than six months [29]. A study conducted in Poland similarly reported that 68.3% of individuals experienced new-onset sleep disturbances after recovery, with 83.6% reporting symptoms that last for more than a month without spontaneous resolution [31]. Although total sleep duration was preserved, many individuals reported irregular sleep patterns like frequent awakenings, difficulty maintaining sleep, moderate to severe insomnia, and increased reliance on sleep aids [31, 32]. The mechanisms underlying poor sleep following COVID-19 infection are likely multifactorial, involving physiological, psychological, and neurological interactions. The acute SARS-CoV-2 infection can trigger inflammatory responses that interfere with neurotransmitter systems responsible for sleep regulation. Additionally, the virus may directly affect the central nervous system (CNS) through hematogenous spread or retrograde neuronal pathways, leading to disruptions in brain regions involved in sleep–wake control [33, 34]. Given these findings, sleep disturbance may serve as an early warning sign of potential long-term consequences following COVID-19 infection. Moreover, sleep disturbances are associated with adverse mental health outcomes, including depression, anxiety, and poor quality of life [35]. The use of self-reported Epworth Sleepiness Scale (ESS) and the Insomnia Severity Index (ISI) could be beneficial for early identification of sleep problems among university students, facilitating timely intervention [31].

Our study found that the number of mRNA COVID-19 vaccine doses received was associated with a 14% reduction in the risk of severe outcomes per additional dose of mRNA (aOR 0.86, 95% CI 0.75–1.00.75.00) among university students. This finding is consistent with a U.S. study, which reported that university students who were either not fully vaccinated or fully vaccinated without a booster had a 2.2–3.0 times higher risk of developing symptoms consistent with long COVID [8]. A systematic review further demonstrated the protective effect of COVID-19 vaccination, particularly with mRNA vaccines, in reducing the incidence of long COVID. The review reported a 15% reduction in odds after one dose (OR 0.85, 95% CI 0.73–0.98, *p* = 0.024), a 24% reduction after two doses (OR 0.76, 95% CI 0.65–0.89), and a 69% reduction after three doses (OR 0.31, 95% CI 0.05–1.84, *p* = 0.198) [36]. While booster vaccinations have shown clear benefits in reducing infection, hospitalization, and death, particularly among older adults and individuals at high risk for severe COVID-19 [37]. Notably, mRNA booster vaccines have been associated with an increased risk of myocarditis, particularly among males aged 18–39 years, with a reported 7.5-fold increased risk within the first week after booster administration. However, most individuals with vaccine-associated myocarditis recovered within 3–8 months after diagnosis [38]. Given these findings, healthcare providers should discuss both the risks and benefits of COVID-19 vaccination with university students, allowing them to make informed decisions about staying up to date with booster doses. The annual booster dose with an mRNA COVID-19 vaccine could be recommended for university students aged 18 years and older, particularly for those at higher risk of severe outcomes from COVID-19 [37]. Individuals who choose not to receive a booster dose should continue to adhere to appropriate health measures to prevent infection.

This study had several limitations. The cross-sectional design precludes the ability to establish causal relationships between potential determinants and severe health outcomes. Recall bias may have occurred, as participants self-reported symptoms that may have changed or improved over time, potentially affecting the accuracy of their responses. Furthermore, the study’s findings may have limited applicability to Thai university students or other undergraduate students with similar ethnic or cultural backgrounds to those in Thailand. Future research should include broader sampling through multicenter designs to capture a more diverse population. Moreover, the use of more robust diagnostic tools to objectively assess Long COVID symptoms would enhance the validity of the findings. Despite these limitations, this study offers valuable insights into the burden of long COVID among university students. The findings could inform stakeholders in developing supportive strategies for early detection, timely treatment, and preventive measures, as well as guiding the creation of university or school policies to address long COVID more effectively.

## Conclusion

A minority of Thai university students reported experiencing long COVID symptoms, most commonly involving the respiratory, neuropsychiatric, and sleep-related symptoms. Receiving additional doses of mRNA COVID-19 vaccines was associated with a reduced likelihood of developing long COVID symptoms, highlighting the potential benefit of booster vaccinations in this population. Early detection and timely intervention, particularly within the first month after COVID-19 diagnosis, are crucial in mitigating serious health outcomes among university students. These findings underscore the need for stakeholders to develop supportive strategies focused on the prevention, early identification, and management of long COVID symptoms. Furthermore, universities and schools should consider implementing guidelines and policies that promote COVID-19 prevention measures, support vaccination uptake, and ensure accessible health services to address the long-term impacts of COVID-19 among young adults.

## Supplementary Information

Supplementary Material 1. Table S1.The Cronbach’s alpha coefficient for each section and subdomain of the questionnaire used in the study.

## Data Availability

The data presented in this study are available on request from the corresponding author.
