# Supplementary material for: The hidden burden: prevalence and risk factors of long COVID among university students in Chiang Mai, Thailand
Source: BMC Public Health. 2025 Nov 24;25:4123. doi: 10.1186/s12889-025-25457-3 (PMC12641946; doi:10.1186/s12889-025-25457-3)
Supplement: Supplementary file 1 — Supplementary Material 1. Table S1.The Cronbach’s alpha coefficient for each section and subdomain of the questionnaire used in the study. [file 12889_2025_25457_MOESM1_ESM.docx]

**Table S1.** The Cronbach’s alpha coefficient for each section and subdomain of the questionnaire used in the study

| **Questionnaire** | **Number of items** | **Cronbach's alpha** | |
| --- | --- | --- | --- |
| **Sleep quality** |  | **Point estimate** | **Bootstrapped 95% CIs*** |
| PSQI part 5.1-5.9 | 9 | 0.870 | (0.854, 0.886) |
| **Organ system symptoms** |  |  |  |
| All systems | 35 | 0.810 | (0.757, 0.863) |
| General | 3 | 0.831 | (0.790, 0.873) |
| Respiratory | 2 | 0.713 | (0.624, 0.802) |
| Cardiovascular | 3 | 0.884 | (0.857, 0.912) |
| Neurologic | 6 | 0.911 | (0.888, 0.935) |
| Psychiatric | 3 | 0.904 | (0.875, 0.933) |
| Gastrointestinal | 3 | 0.878 | (0.826, 0.931) |
| Skin | 3 | 0.747 | (0.678, 0.816) |
| ENT | 4 | 0.871 | (0.823, 0.919) |
| Musculoskeletal | 5 | 0.885 | (0.852, 0.917) |
| Immunology | 3 | 0.810 | (0.757, 0.863) |
| **Behavior** |  |  |  |
| 20-item behavior | 20 | 0.942 | (0.936, 0.949) |

* Bootstrapped was carried out using 1000 sampling-resampling across the entire samples
